# Conclusions from the symposium. Two decades of ART: success through research

**DOI:** 10.1590/S1678-77572009000700021

**Published:** 2009

**Authors:** Christopher J. HOLMGREN, Jo E. FRENCKEN

**Affiliations:** 1 BDS, FDSRCS, PhD, Department of Global Oral Health, College of Dental Sciences, Radboud University Nijmegen Medical Centre, The Netherlands.; 2 DDS, MSc, PhD, Department of Global Oral Health, College of Dental Sciences, Radboud University Nijmegen Medical Centre, The Netherlands.

**Keywords:** Atraumatic Restorative Treatment (ART), Dental caries, Health services research

## Abstract

Two decades of ART research has served as the catalyst for a new way of thinking about oral health care. It is now necessary to build on the success of ART research by educating existing and future oral health professionals and health decision makers about the benefits of the ART approach. It is also important to build upon the sound research base that already exists on ART even though enough is known about ART to consider it is a reliable and quality approach to control caries. While oral health promotion through prevention remains the essential foundation of oral health, the ART approach is an important corner stone in the building of global oral health.

## INTRODUCTION

While it might seem only yesterday for those who pioneered the development and research of the Atraumatic Restorative Treatment (ART) approach, two decades have already passed since the start of the first major study on ART in Khonkaen, Thailand^3^. The timing of this present symposium therefore serves not only as a temporal milestone for ART but also marks the principal outcome of two decades of ART research, namely its contribution to successfully improving oral health worldwide.

In organizing this important symposium, reviewing and building upon the two decades of ART research, it was only appropriate that it be held in Latin America, for although ART did not originate there, it is a Region where huge attention has been focused on the ART approach. It is also a Region which has in many ways been at the forefront recently in many fields of ART, both from a research perspective and in its application of ART in community and country programs as is evidenced by the presentations at this symposium.

Over ten years have passed since the last international ART symposium devoted to research during the IADR congress in Nice in 1998^7^. The proposal by Dr. Olga Zambrano (Venezuela) and Dr. Márcia Cançado Figueiredo (Brazil), that this current symposium be held during the 3^rd^ IADR Congress of the Latin American Region in Isla Margarita, Venezuela, November 2009, provided a timely opportunity to take stock of twenty years of research and build on success by acting as a springboard for future ART research. It also provided an opportunity to bring together prominent researchers in the field of ART in Latin America. Here, we were delighted that the symposium speakers, representing different fields of research from four different countries in the Latin American Region were able to participate and provide extremely valuable contributions both to the symposium itself and to a wider audience through the eventual publication of these proceedings. We were also particularly pleased that the President elect of IADR, Dr Fidela Navarro was able to present at the symposium.

## EARLY RESEARCH

In the early 1990's, research into the ART approach was spearheaded by a few dedicated workers who saw the true potential for this approach. This research was neither easy nor straightforward since it was often conducted under difficult conditions in the field on shoestring budgets. Moreover, such research was often not appreciated or valued by our peers since ART challenged traditional concepts of restorative treatment and caries management. Despite the early resistance by many to the ART approach, some of whom considered ART to be “third-world dentistry” or “dentistry out of Africa” or even “dirty dentistry”, time has proven such pundits wrong. This was achieved through a combination of sound research to provide an excellent evidence base for the approach and logical common sense. Through this approach oral health care can readily be transported and used in any setting making care more accessible to the many thousands of millions who do not have ready access to care.

## INTERNATIONAL ACCEPTANCE

While research has provided the evidence base, the worldwide awareness of the ART approach can be attributed to the substantial support from other sectors. The extremely encouraging results of the first ART studies led to support of the approach by international health organisations including the World Health Organisation (WHO)^9^, the FDI World Dental Federation^8^, the IADR^6^^,^^7^, and later the Pan American Health Organisation (PAHO)^5^. This latter organisation, through funding of the Inter-American Development Bank (IDB), also organised Project PRAT, a study whose main objective was to demonstrate the cost-effectiveness of the ART approach in a variety of settings in the Region in comparison to the cost- effectiveness of the amalgam technique in the same settings^5^. Despite the numerous problems encountered during the study in which we provided considerable input in training, methods and study design, it was an important first-step to further and perhaps better controlled cost-effectiveness studies in the future.

## RESEARCH OUTCOMES

As is evidenced by the papers presented during this symposium, over the past twenty years ART has become one of the most researched approaches for the control of dental caries and certainly for minimal intervention (MI) approaches for caries^1^^,^^2^. In this respect ART could be considered in many ways to be the spearhead of MI. It certainly helped to build the momentum of the MI movement amongst a traditionally conservative dental profession who are often slow to grasp new approaches, even those that have a significant evidence base, and adopt them as part of their day-to-day practice armamentarium.

While the past two decades of ART can be heralded as a success story, it is necessary to build on this success. One part of this is to educate existing and future oral health professionals and health decision makers about the benefits of the ART approach. The other is to build upon the sound research base that already exists on ART. While we know enough about ART to know it is a reliable and quality approach to control caries, there will always be a call for addition research to improve on success. In particular, in order to make the quantum leap forward to achieve a significant improvement in oral health in all countries of the world there will be a need for resources to be allocated to applied research on approaches such as ART and allied areas. Here such research is hindered by a lack of funding, a lack of motivated and capable researchers often plagued by the publish or perish syndrome^4^. Furthermore, such research sometimes lacks recognition by the research community and in some cases it is difficult to publish such research, the latter because of the lack of understanding of the difficulties involved in conducting applied research under real life circumstances. This having been said, the more than 170 publications dealing with the ART approach bear witness to the motivated and capable researchers who have often put oral health research ethics before personal gain.

## CONCLUSIONS

In summing up the last two decades of ART research, ART has served as the catalyst for a new way of thinking about oral health care. While oral health promotion through prevention remains the essential foundation of oral health, the ART approach is an important corner stone in the building of global oral health. We wish to thank all those who have contributed through their research to making the world a healthier and happier place.

## References

[1] Dawson AS, Makinson OF (1992). Dental treatment and dental health. Part 1. A review of studies in support of a philosophy of Minimum Intervention Dentistry. Aust Dent J.

[2] Dawson AS, Makinson OF (1992). Dental treatment and dental health. Part 2. An alternative philosophy and some new treatment modalities in operative dentistry. Aust Dent J.

[3] Frencken JE, Songpaisan Y, Phantumvanit P, Pilot T (1994). An Atraumatic Restorative Treatment (ART) technique: evaluation after one year. Int Dent J.

[4] Katchburian E (2008). Publish or perish: a provocation. São Paulo Med J.

[5] Pan American Health Organization. Division of Health Systems and Services Development (2006). Oral health of low income children: procedures for Atraumatic Restorative Treatment (PRAT). Final Report.

[6] (1996). Proceedings of the IADR symposium “Minimal intervention techniques for dental caries”. J Public Health Dent.

[7] (1999). Proceedings of the IADR symposium “The state of ART (Atraumatic Restorative Treatment) - a scientific perspective”. Community Dent Oral Epidemiol.

[8] Tyas MJ, Anusavice KJ, Frencken JE, Mount GJ (2000). Minimal intervention dentistry - a review. FDI Commission Project 1-97. Int Dent J.

[9] World Health Organization (1994). Revolutionary new procedure for treating dental caries.

